# Surgical Treatment of Adrenal Neuroblastoma in Children – A Narrative Review

**DOI:** 10.1590/S1677-5538.IBJU.2026.0168

**Published:** 2026-05-04

**Authors:** Arovel Oliveira Moura, Francisca Norma Albuquerque Girão Gutierrez, Fádia Carvalho Pacheco, Ricardo Vianna de Carvalho, Ruy Garcia Marques

**Affiliations:** 1 Instituto Nacional de Câncer – INCA Cirurgia Pediátrica Oncológica Rio de Janeiro RJ Brasil Cirurgia Pediátrica Oncológica, Instituto Nacional de Câncer – INCA, Rio de Janeiro, RJ, Brasil; 2 Instituto Nacional de Câncer – INCA Informação Científica e Tecnológica em Saúde Rio de Janeiro Brasil Informação Científica e Tecnológica em Saúde, Instituto Nacional de Câncer – INCA, Rio de Janeiro, Brasil; 3 Universidade do Estado do Rio de Janeiro Faculdade de Ciências Médicas Departamento de Cirurgia Geral Rio de Janeiro RJ Brasil Departamento de Cirurgia Geral, Programa de Pós-graduação em Fisiopatologia e Ciências Cirúrgicas, Faculdade de Ciências Médicas – Universidade do Estado do Rio de Janeiro - UERJ, Rio de Janeiro, RJ, Brasil

**Keywords:** Neuroblastoma, Adrenal Glands, Surgical Procedures, Operative

## Abstract

**Objective:**

To analyze the role of surgical treatment in pediatric adrenal neuroblastoma and the operative methods employed according to staging and risk stratification based on patient-related and tumor biological factors.

**Materials and Methods:**

A narrative literature review was conducted through a structured search of the PubMed/MEDLINE, Scopus, and Web of Science databases. Studies published in English between 2020 and 2025 were included, while classical references were used to contextualize staging and risk stratification systems. Letters, editorials, case reports, and studies not providing specific insight into the topic were excluded.

**Results:**

Surgical treatment represents a key component in the management of pediatric adrenal neuroblastoma. Staging and risk stratification determine both the timing of surgery and its role in achieving local disease control. In low-risk neuroblastoma, surgery alone may be curative. In intermediate-risk patients, neoadjuvant chemotherapy precedes resection, reducing surgical complexity and facilitating tumor removal. In high-risk patients, tumor resection exceeding 90% may contribute to improved overall survival (OS). Image-Defined Risk Factors (IDRFs) are essential for staging and surgical planning, as they define the anatomical relationship between the tumor and adjacent vascular and organ structures. Surgical approaches include open surgery and minimally invasive surgery, the latter being appropriate in carefully selected cases.

**Conclusion:**

Surgical treatment remains fundamental in pediatric adrenal neuroblastoma, and operative strategies should be guided by clinical, biological, and radiologic parameters to achieve maximal oncologic safety.

## INTRODUCTION

Neuroblastoma (NB) is the most common extracranial solid malignancy in children, with an annual incidence rate of 13.2 per 1,000,000 children per year. It originates from neural crest cells. Its most frequent location is the adrenal medulla. Bilateral presentation is rare and is generally associated with a worse prognosis compared to NB located in other anatomical sites along the paravertebral sympathetic chain. The biological heterogeneity of NB influences spontaneous regression, prognosis, and the possibility of cure (1–3).

Nong et al. (2025), in a comprehensive global epidemiological study on NB in the pediatric population, concluded that despite fluctuations in trends of incidence, mortality, and disability-adjusted life years (the sum of years of life lost due to premature death and years lived with disability), the disease burden remains considerable in regions with low, lower-middle, and middle socioeconomic development indices (4).

An important retrospective study conducted by Zhang et al. (2023) demonstrated a higher prevalence of adrenal NB in male children (56.0%), right-sided unilateral location (66.7%), and tumor size greater than 4 cm (85.3%), with most cases presenting in advanced stages and with distant metastases. For radiologic diagnosis, computed tomography proved to be the method of choice compared with ultrasonography (p < 0.05) (5).

For nearly five decades, criteria for NB staging have been established. The Children's Cancer Group (CCG), a North American cooperative group, developed and updated staging systems, including the International Neuroblastoma Staging System (INSS) and the International Neuroblastoma Risk Group Staging System (INRGSS) (6, 7).

Risk stratification guides the treatment of these patients. Research strategies dedicated to understanding the biological profile of NB have contributed to increasingly precise risk classification, resulting in therapeutic approaches proportional to tumor complexity (8).

The role of the pediatric oncologic surgeon in adrenal NB ranges from obtaining tissue for diagnosis to performing tumor resection with therapeutic intent and has been progressively influenced by the transition from the INSS system to the INRGSS, as well as by the consolidation of minimally invasive techniques. Spencer et al. (2022), in a 20-year retrospective analysis, demonstrated a significant reduction in open biopsies after 2009 (45% vs. 26%, p = 0.04), reflecting greater incorporation of percutaneous methods. However, the increase in minimally invasive surgeries (MIS) during the same period did not reach statistical significance (3% vs. 9%, p = 0.14), suggesting that changes in diagnostic practice preceded definitive consolidation of minimally invasive approaches in surgical treatment (9).

In low-risk adrenal NB, treatment strategies range from observation to surgical resection aimed at complete removal of the tumor mass and regional lymph nodes. Chemotherapy is typically reserved for patients with adverse biological characteristics. Contemporary risk-adapted management has resulted in a 5-year OS of nearly 98%. (10).

In intermediate-risk adrenal NB (INRG L2 with IDRFs), without MYCN amplification, neoadjuvant chemotherapy aims to reduce tumor volume and technical complexity of resection, with duration and intensity adjusted according to clinical response and tumor biological factors. After satisfactory response, complete macroscopic resection is pursued. Partial resection may be acceptable when IDRFs persist involving major vessels or critical structures and when radicality would imply disproportionate morbidity, considering that oncologic control in this group is predominantly systemic (11, 12).

The treatment of high-risk adrenal NB is multimodal, intensive, and sequential. It begins with systemic induction chemotherapy, during which surgical resection of the primary tumor is performed after adequate volumetric reduction. During induction, hematopoietic stem cell collection is also carried out for subsequent autologous transplantation. The consolidation phase includes high-dose chemotherapy followed by autologous bone marrow transplantation and external beam radiotherapy directed to the tumor bed and any residual metastatic sites. Finally, the post-consolidation phase includes immunotherapy based on anti-GD2 antibody combined with isotretinoin, a strategy that has demonstrated significant gains in event-free survival (EFS) and OS (13, 14).

Favorable surgical outcomes following systemic disease control are reported in a South African study conducted by van Heerden et al. (2022), which included 204 high-risk NB patients, 105 of whom underwent surgery and 82 with adrenal tumors. In univariate prognostic analysis, patients who achieved remission of metastatic disease — 45/56 (80.4%) — had their tumors resected (p < 0.001) (15).

Pediatric adrenalectomy may be performed via open surgery or, in selected cases, by minimally invasive surgery (MIS), including transperitoneal laparoscopic adrenalectomy (TLA), posterior retroperitoneoscopic adrenalectomy (PRA), and robot-assisted surgery. TLA is the most widely adopted approach and is considered safe in children without IDRFs, particularly in small, well-delimited tumors without vascular invasion. The choice of approach should be based on tumor size, presence of IDRFs, and relationship with vascular structures, reserving open surgery for extensive lesions or those with significant vascular involvement (16, 17). Zhong et al. (2021) reported that PRA is a feasible and safe surgical approach for the management of pediatric adrenal oncologic diseases, including NB. In this comparative study between TLA and PRA, the authors primarily assessed pathological outcomes, operative time, transfusion requirements, conversion to open surgery, time to resumption of oral intake, and length of hospital stay (18).

Van Uitert et al. (2025) published a Dutch national multicenter study (2011–2022) including 137 pediatric patients who underwent adrenalectomy, of whom 87 (64%) had suspected NB. Among the 86 histologically confirmed NB cases, open surgery was used in 73 patients (85%), whereas 13 (15%) underwent TLA, with no cases treated by PRA (19).

Considering the marked biological heterogeneity and consequent diversification in the clinical presentation of adrenal NB in children, the objective of this narrative review is to highlight the role of surgical techniques employed, considering risk stratification systems based on patient-related factors and tumor biological characteristics.

## MATERIALS AND METHODS

This narrative review aimed to provide a comprehensive and updated analysis of the evidence regarding the surgical treatment of primary adrenal NB in pediatric patients. A structured literature search was conducted to support the thematic discussion; however, the study design followed an interpretative and integrative approach, consistent with the narrative review model.

The search was performed in the PubMed/MEDLINE, Scopus, and Web of Science databases, including publications indexed within the last six years (2020–2025). The strategy combined controlled descriptors (DeCS/MeSH): "Neuroblastoma," "Adrenal Glands," "Surgical Procedures, Operative," and "Adrenalectomy." Additionally, a manual review of the reference lists of selected studies was conducted to identify potentially relevant publications not captured by the initial electronic search strategy.

Although the structured database search supported the identification of contemporary studies, additional publications were included based on their clinical relevance, cooperative group guidelines, and contributions considered fundamental for contextualizing staging systems and surgical decision-making models. This approach is consistent with the interpretative and integrative nature of narrative reviews.

Given the narrative design of this study, no formal systematic review protocol, quantitative synthesis, or PRISMA flow diagram was applied. Study selection was guided by thematic relevance and clinical applicability to the proposed conceptual and surgical axes.

Eligible studies included those involving pediatric patients that addressed aspects related to surgical strategy, operative timing (primary surgery or post-neoadjuvant chemotherapy), extent of tumor resection, perioperative morbidity, or oncologic outcomes. Multicenter cooperative studies were also included, given their relevance in standardizing risk stratification and guiding therapeutic decisions.

Excluded were letters to the editor, editorials, case reports, studies focused exclusively on extra-adrenal NBs, duplicate publications, and articles published in languages other than English.

Data were organized through qualitative thematic synthesis, structured according to analyses related to surgical indication, operative planning based on radiologic risk factors, degree of resection radicality, role of minimally invasive approaches, operative morbidity, and prognostic impact.

## RESULTS

With the evolution of multimodal systemic therapies, the isolated relevance of surgery in NB has decreased, without eliminating its therapeutic necessity. Pediatric oncologic surgeons are responsible for defining the therapeutic strategy based on a correct understanding of tumor biological behavior, while maintaining the perspective of improved prognosis (20).

Tumors located in the adrenal gland may be classified into two major groups: adrenocortical tumors — including adenoma and adrenocortical carcinoma — and tumors of the adrenal medulla, such as NB and pheochromocytoma (21).

In the presence of clinical and/or laboratory suspicion of a cortical tumor or pheochromocytoma, tumor biopsy should generally be avoided due to potential hormonal repercussions and hemodynamic complications. Conversely, in suspected NB, tumor biopsy is recommended for diagnostic purposes, since histological analysis and molecular characterization of the tumor are prerequisites for adequate risk stratification and therapeutic definition (22).

Historically, NB staging was initially structured according to the INSS (Table-1), later replached by the INRGSS (Table-2), which incorporated preoperative criteria based on radiologic and biological factors. Recent advances in research have been directed toward the identification and validation of genes associated with tumor staging according to the INSS, highlighting their potential as biomarkers in prognostic models for NB. (6, 7, 23).

**Table 1:** Staging according to the International Neuroblastoma Staging System (INSS).

INSS	Definition	Key Characteristics
1	Localized tumor with complete gross excision, with or without microscopic residual disease; ipsilateral lymph nodes negative.	Complete resection; negative LNs
2A	Localized tumor with incomplete gross excision; ipsilateral lymph nodes negative.	Incomplete resection; negative LNs
2B	Localized tumor with or without complete excision; ipsilateral lymph nodes positive; contralateral negative.	Ipsilateral positive LNs
3	Tumor crossing the midline or unilateral tumor with contralateral regional lymph node involvement.	Midline crossing or contralateral LNs
4	Primary tumor with distant metastasis (except stage 4S).	Distant metastasis
4S	Localized primary tumor with limited metastasis to skin, liver, and/or bone marrow in infants <1 year.	Localized + limited metastasis (<1 year)

**Table 2:** Staging according to the International Neuroblastoma Risk Group Staging System (INRGSS).

INRGSS	Definition	Key Characteristics
L1	Locoregional tumor without image-defined risk factors (IDRFs).	Localized; no IDRFs
L2	Locoregional tumor with one or more IDRFs.	Localized; IDRFs present
M	Distant metastatic disease.	Metastasis (except MS)
MS	Metastatic disease confined to skin, liver, and/or bone marrow in patients <18 months.	Infants <18 months

Risk grouping depends on demographic (age at diagnosis) and clinical factors (stage, MYCN gene amplification, histopathology, chromosomal aberrations, and cellular ploidy). Proper staging and accurate risk stratification lead to treatment strategies associated with improved outcomes (Table-3) (12, 24).

**Table 3:** Patient-related and tumor biological factors associated with risk stratification.

Risk Factor	Type	Relevance and Impact
Age at diagnosis	Demographic	Diagnosis <18 months: lower risk; >18 months: higher risk, reduced EFS.
MYCN amplification	Cytogenetic	Associated with poor prognosis and high-risk disease.
Ploidy	Cytogenetic	Diploid tumors associated with worse outcomes.
Chromosomal alterations	Cytogenetic	17q gain; 1p/11q deletion linked to aggressive disease.
Histology	Morphological	Poor differentiation associated with high risk.

EFS = Event-Free Survival

Liukitithara et al. (2022) reported a cohort of 37 pediatric patients (2007–2016), 19 of whom had adrenal NB (51.3%), with 73.0% classified as high risk and nearly all (18/19) presenting with INSS stage IV and metastatic disease, reflecting late diagnosis (21). Similar findings were reported by Khan and Verma (2025) in a cohort of 53 patients (2016–2022), including 31 (58.5%) with adrenal NB. Among those staged according to INRGSS (49/53), more than half were diagnosed at M or MS stages, indicating metastatic disease (25).

Certain critical elements related to adrenal NB surgery deserve particular attention. When technically feasible and safe, resection exceeding 90% of tumor volume is recommended, extending to the limits of contiguous structures, with preservation of major involved vessels. En bloc resection of adjacent organs is not routinely recommended. Lymph node dissection should include the renal pedicle chains, azygos vein chains, intercavoaortic chains, and pericaval chains. Above all, the primary recommendation is preservation of patient integrity (26, 27).

An important biological tumor factor to be evaluated is MYCN gene amplification in tumor tissue obtained by diagnostic biopsy. When gene amplification is present, recommended surgical resection often becomes unfeasible at presentation. Neoadjuvant chemotherapy aims to reduce tumor volume, minimize the effects of proximity to critical structures, decrease the risk of injury to vital structures, and optimize surgical outcomes (28).

To expand surgical understanding through standardized data collection, pediatric surgeons from cooperative pediatric oncology groups developed the first detailed and structured surgical report for NB, including adrenal tumors, under the premise of establishing an international consensus adaptable to different local realities (29).

Tumor volume represents an important predictive prognostic factor, with tumors larger than 4 cm associated with unfavorable evolution (30). Conversely, in neonates and infants — an age group generally associated with better prognosis — serial magnetic resonance imaging may justify initial nonoperative management, known as a "watch-and-wait" strategy (31).

**Figure 1:**
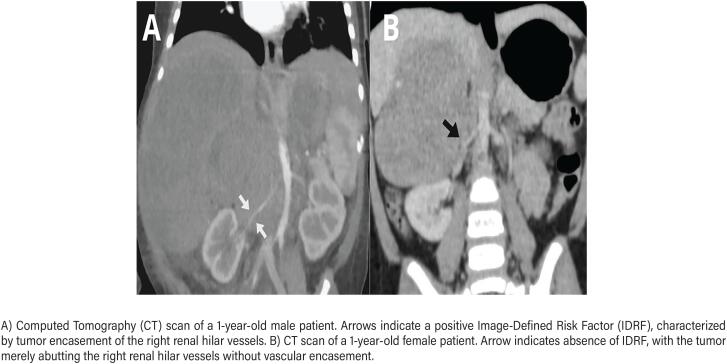
Radiological aspects of adrenal neuroblastoma.

Identification of IDRFs through computed tomography or magnetic resonance imaging constitutes a central element in assessing resectability of adrenal NB (Figure-1). These radiologic parameters reflect the anatomical relationship between the tumor and critical adjacent structures, particularly major abdominal vessels — such as the aorta and inferior vena cava — as well as the celiac axis and renal pedicle. The presence of IDRFs is associated with greater technical complexity and lower likelihood of complete macroscopic resection, whereas their absence facilitates extensive tumor excision with oncologic safety. Under such circumstances, unresected tumors are associated with worse oncologic prognosis (Table-4) (32–34).

A) Computed Tomography (CT) scan of a 1-year-old male patient. Arrows indicate a positive Image-Defined Risk Factor (IDRF), characterized by tumor encasement of the right renal hilar vessels. B) CT scan of a 1-year-old female patient. Arrow indicates absence of IDRF, with the tumor merely abutting the right renal hilar vessels without vascular encasement.

**Table 4:** Image-Defined Risk Factors (IDRFs) related to adrenal topography.

IDRF Attribute	Radiological Definition and Criteria
Contact	Absence of a visible fat plane between the tumor and adjacent normal tissue and/or involvement of <50% of the vessel circumference.
Encasement	Absence of a visible fat plane between the tumor and the adjacent vessel with >50% circumferential arterial involvement. Venous flattening and obliteration of the vascular lumen may be observed.
Infiltration	Tumor extension into adjacent organs. Direct involvement of the renal artery, renal vein, or renal pelvis is technically classified as renal invasion.

A systematic review and meta-analysis by Parhar et al. (2020) aimed to quantify the predictive capacity of IDRFs assessed at pretreatment (before neoadjuvant chemotherapy) in relation to surgical outcomes and patient survival. The authors concluded that the presence of IDRFs increased the risk of incomplete surgical resection and postoperative complications, as well as reduced five-year EFS and OS (35).

Espinoza et al. (2024), in a multicenter study, demonstrated a lower probability of complete resection (>90%) in children with a higher number of IDRFs, both at diagnosis and at the time of surgical treatment (p = 0.0004) (36). In contrast, a single-center study by Liu et al. (2024) demonstrated that the number of IDRFs significantly decreased after four cycles of neoadjuvant chemotherapy (p = 0.034). The study showed that both the quantity and quality of preoperative IDRFs may predict secondary surgical outcomes beyond simple presence or absence (37).

With the aid of information technology, using radiomics-based machine learning (application of advanced algorithms to extract large quantitative datasets from medical images), surgical risk in children with adrenal NB may be predicted based on criteria including major vascular involvement (celiac axis, superior mesenteric artery, iliac vessels, inferior vena cava), tumor crossing the midline, retroperitoneal lymph node metastasis, renal pedicle invasion, and hepatic, pancreatic, or duodenal invasion (38).

Avanzini et al. (2025), in a collaborative study, stratified and assigned relevance scores to each IDRF using data collected between 2016 and 2020, correlating them with surgical risks. The study aimed to provide more precise definitions of surgical objectives and ideal conditions favoring macroscopic tumor resection (39).

In high-risk adrenal NB, surgery is incorporated during the induction phase, with a possible role also during consolidation. OS and EFS appear to be positively impacted by significant tumor resections (>90%) (13, 14, 40).

In rare cases of bilateral adrenal NB, a more conservative surgical approach on the less affected side should be considered. Prognosis in these patients is more dependent on disease extent and tumor biological profile, such as MYCN gene amplification (40).

Anesthetic management, an integral component not only of the operative procedure but also of tumor biopsy and postoperative pain control, requires thorough preoperative clinical evaluation. Anesthetic considerations depend largely on tumor location, operative approach, and extent of surgical dissection. Particular attention should be given, even in previously healthy patients, to tumor- or chemotherapy-induced physiopathological alterations affecting cardiovascular, gastrointestinal, and immune systems (42).

**Figure 2:**
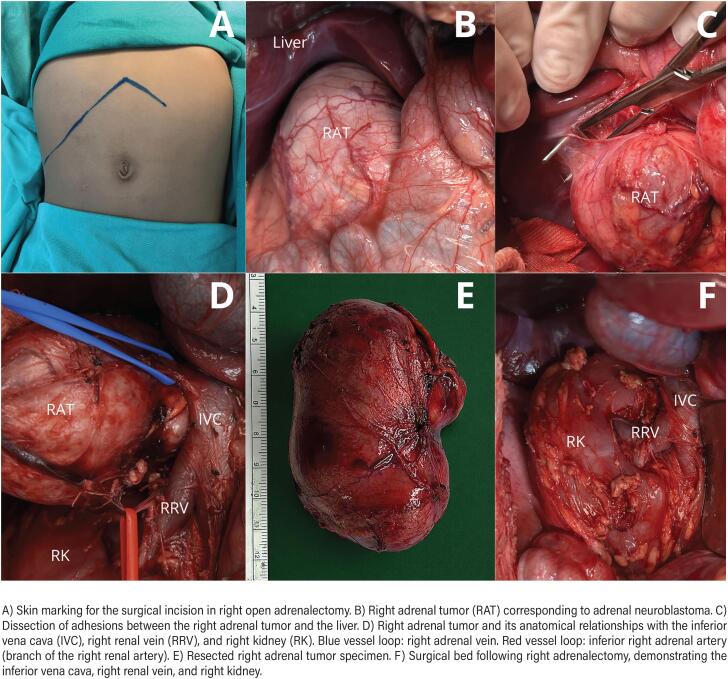
3-year-old male patient.

Open transperitoneal surgery has been employed for decades as an alternative to excise adrenal NB infiltrating adjacent tissues — especially vessels or lymph nodes — and tumors crossing the midline. Incisions may be combined according to the required exposure when the tumor extends superiorly or medially. This surgical technique remains a solid strategy to achieve complete tumor excision (Figure-2) (43).

A) Skin marking for the surgical incision in right open adrenalectomy. B) Right adrenal tumor (RAT) corresponding to adrenal neuroblastoma. C) Dissection of adhesions between the right adrenal tumor and the liver. D) Right adrenal tumor and its anatomical relationships with the inferior vena cava (IVC), right renal vein (RRV), and right kidney (RK). Blue vessel loop: right adrenal vein. Red vessel loop: inferior right adrenal artery (branch of the right renal artery). E) Resected right adrenal tumor specimen. F) Surgical bed following right adrenalectomy, demonstrating the inferior vena cava, right renal vein, and right kidney.

Fiori et al. (2020) recommend laparoscopy as first choice for benign adrenal tumors measuring 6–8 cm and possibly extending to 10–12 cm in the absence of radiologic signs of malignancy. Extension of traditional size limits has been associated with favorable outcomes in centers with specific expertise in minimally invasive adrenal surgery (44).

Uttinger et al. (2022), in a retrospective analysis of pediatric adrenalectomies in Germany (2009–2017), reported a prevalence of NB (56%) among malignant adrenal tumors, predominantly in children younger than five years. The number of open surgeries exceeded MIS in all participating hospitals, regardless of surgical volume (p = 0.015) (45).

Initially, MIS was used for diagnostic procedures and subsequently extended to tumors such as adrenal NBs. The same oncologic principles of open surgery — negative margins, prevention of tumor rupture, and extensive lymphadenectomy when indicated — must be replicated in MIS to ensure excellent oncologic outcomes (46).

**Figure 3:**
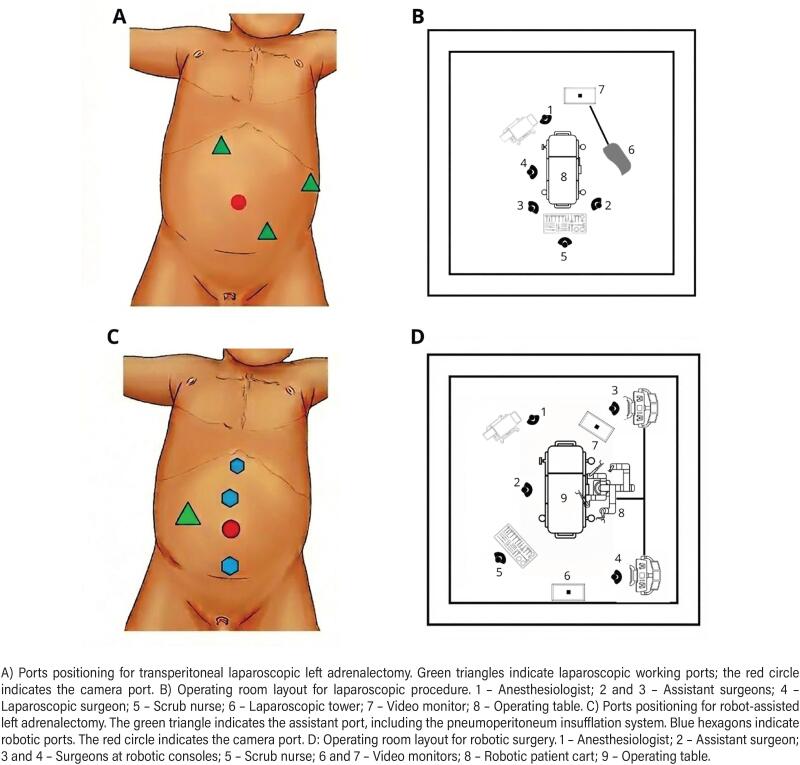
Minimally Invasive Surgery (MIS) setup for adrenal neuroblastoma resection.

The main laparoscopic approaches include transperitoneal and posterior retroperitoneoscopic techniques. The transperitoneal route is preferred due to anatomical familiarity, wider operative field, and easier simultaneous abdominal exploration. The surgical setup and trocar positioning are illustrated in Figure-3 (A and B). The posterior retroperitoneoscopic approach allows direct gland access without violation of the peritoneal cavity, reducing postoperative pain, operative time, and hospital stay, albeit with a more restricted operative field (17).

A) Ports positioning for transperitoneal laparoscopic left adrenalectomy. Green triangles indicate laparoscopic working ports; the red circle indicates the camera port. B) Operating room layout for laparoscopic procedure. 1 – Anesthesiologist; 2 and 3 – Assistant surgeons; 4 – Laparoscopic surgeon; 5 – Scrub nurse; 6 – Laparoscopic tower; 7 – Video monitor; 8 – Operating table. C) Ports positioning for robot-assisted left adrenalectomy. The green triangle indicates the assistant port, including the pneumoperitoneum insufflation system. Blue hexagons indicate robotic ports. The red circle indicates the camera port. D: Operating room layout for robotic surgery. 1 – Anesthesiologist; 2 – Assistant surgeon; 3 and 4 – Surgeons at robotic consoles; 5 – Scrub nurse; 6 and 7 – Video monitors; 8 – Robotic patient cart; 9 – Operating table.

Regarding robotic surgery, increasing evidence suggests that its structural advantages translate into intraoperative technical benefits and consequently faster postoperative recovery and potentially lower morbidity. The surgeon operates from a dedicated robotic console, which enables precise control of articulated instruments and high-definition three-dimensional visualization of the surgical field. The robotic surgical setup adopted for adrenal tumor resection is illustrated in Figure-3 (C and D). Relative contraindications include the presence of one or two IDRFs. Formal contraindications include more than two IDRFs or any number involving unpaired vessels — such as the celiac artery or superior mesenteric artery — and/or bilateral renal pedicle involvement (47, 48).

As highlighted by Matthyssens et al. (2021), standardization of complication reporting through the International Neuroblastoma Surgical Report Form (INSRF) reduces variability in operative documentation and allows more consistent comparison among surgical strategies. Complications within 30 postoperative days are classified according to the Clavien–Dindo system, ranging from grade I (minor interventions) to grade V (death) (29).

Surgical morbidity remains a critical factor in defining the ideal extent of resection in pediatric adrenal NB, particularly in tumors with vascular involvement. Perioperative complications such as lymphatic leakage, hypertension, massive hemorrhage, important structural injury, inadvertent resection of non-infiltrated organs and minor complications (hypoglycemia, intestinal obstruction, abdominal infection, incision-related complication) reflect this technical complexity. These events may compromise functional preservation, delay continuation of multimodal therapy, and negatively impact oncologic outcomes (49).

Chylous ascites represents a relevant complication following adrenal NB resection due to the intimate anatomical relationship with retroperitoneal lymphatic channels and the thoracic duct pathway (50). Froeba-Pohl et al. (2020), in a cohort of 204 patients, 98 (48%) with adrenal NB, reported lymphatic leakage in 40% of cases, correlated with greater extent of resection (p = 0.005), advanced stage (p = 0.0001), and MYCN amplification (p = 0.019) (51).

Postoperative pleural effusion is another significant complication following adrenal NB resection. Hu et al. (2025) reported an incidence of 21.8%. Adrenal origin (aOR = 16.20; p = 0.035), operative time ≥ 4.33 hours (aOR = 180.20; p = 0.001), and early hypoalbuminemia (aOR = 17.13; p < 0.001) were independent predictors. These findings suggest that the complication is predominantly related to surgical trauma magnitude and systemic inflammatory response (52).

Alikärri et al. (2023), in a systematic review, demonstrated that sympathetic denervation resulting from subadventitial vessel resection in NB involving the superior mesenteric artery may cause intestinal parasympathetic hyperactivity, accelerating bowel transit. This diarrhea, distinct from that associated with excessive vasoactive intestinal peptide secretion by NB, is typically prolonged and requires pharmacologic and dietary management (53).

Laparoscopic and robot-assisted approaches have demonstrated feasibility and safety in localized tumors, with perioperative benefits such as reduced blood loss and shorter hospital stay. However, consistent evidence of oncologic superiority over open surgery is lacking, reinforcing the need for individualized indication based on strict anatomical criteria. Major studies evaluating MIS in pediatric adrenal NB are summarized in Table-5. Predominantly, these are retrospective, single-center series with carefully selected patients, generally without IDRFs (46, 54–62).

**Table 5:** Studies evaluating minimally invasive surgery (MIS) for adrenal neuroblastoma in children.

Author	Study Design	Population	Objective	Method	Results / Conclusion
Oesterreichet al. (2022)(54)	Retrospective, descriptive, single center	28 patients; 17 adrenal NB (60.7%)	Analyze role of laparoscopic adrenalectomy (LA)	Retrospective MIS analysis (2003–2020)	LA is safe for biopsy when percutaneous access is not feasible; appropriate for localized small-volume tumors.
Blanc et al. (2022) (46)	Prospective, multicenter	100 tumors; 12 adrenal NB (12%)	Evaluate first 100 robotic procedures	Feasibility and safety study (2016–2020)	Robotic surgery is feasible and safe; 8% conversion rate; no adrenal NB complications.
Chang et al. (2023) (55)	Retrospective, comparative, single center	87 abdominal NB without IDRFs; 67 adrenal (77.0%)	Assess laparoscopic safety and efficacy	Open (54) vs LA (33), 2016–2021	Less blood loss (p=0.013) and earlier feeding (p=0.002); no oncologic difference.
Sugita et al. (2023) (56)	Retrospective, comparative, single center	22 patients; 19 adrenal NB (86.3%)	Compare biopsy and curative resection	Surgical/histopathological comparison	Laparoscopy feasible and safe; open biopsy shorter operative time (p=0.02).
Pio et al. (2023) (57)	Retrospective, comparative, single center	33 patients; 25 adrenal NB (75.7%)	Compare PR vs TP approach	Perioperative analysis	PR approach reduced opioid use (p=0.02) and hospital stay (p=0.008).
Kawakubo et al. (2024) (58)	Retrospective, single center	24 peripheral neuroblastic tumors; 11 adrenal NB	Clarify endoscopic indications	Preoperative CT IDRF assessment (2007–2022)	Safe without IDRFs; IDRF status crucial.
Mora Fritis et al. (2024) (59)	Retrospective, observational, single center	14 patients; 7 adrenal NB (50%)	Report on MIS outcomes	Observational review (2012–2023)	MIS safe; no intraoperative mortality; mean stay 1 day.
Tian et al. (2024) (60)	Retrospective, descriptive, single center	27 patients; 16 adrenal NB (59.2%)	Describe single-incision PR technique	Technical evaluation (2020–2023)	Requires expertise; excellent postoperative outcomes.
Taghavi et al. (2024) (61)	Prospective, observational, single center	39 robot-assisted cases; 23 NT, 20 adrenal NB	Evaluate robot-assisted adrenalectomy (RAA)	Six-year prospective study	RAA safe and effective; macroscopically negative margins achieved.
Liu et al. (2025) (62)	Retrospective, comparative, single center	22 adrenal NB (12 LA; 10 robotic)	Compare robotic vs laparoscopic	Retrospective analysis (2012–2024)	Robotic group: lower blood loss (p=0.036), shorter drainage (p=0.031), shorter stay (p=0.021)

NB = Neuroblastoma; MIS = Minimally Invasive Surgery; LA = Laparoscopic Adrenalectomy; PR = Posterior Retroperitoneoscopic; TP = Transperitoneal; IDRFs = Image-Defined Risk Factors; RAA = Robot-Assisted Adrenalectomy; NT = Neuroblastic Tumor

Regarding prognosis, surgical resection is associated with improved oncologic outcomes, particularly in children with NB. Even in high-risk adrenal NB, improvements in one-, three-, and five-year OS are observed in patients undergoing surgical resection. Late diagnosis and large tumors with distant metastases represent independent risk factors when analyzing cancer-specific survival (CSS) (63, 64). A nomogram proposed by Zheng et al. (2020), supporting predictive clinical analysis, proved consistent in evaluating OS and CSS in children undergoing surgery for adrenal NB (65).

As synthesized in Table-6, studies reinforce that surgical treatment should be understood as a strategic component of multimodal therapy, with individualized indication based on the biological profile of the disease, including MYCN amplification, age at diagnosis, and risk category. Importantly, these studies consistently demonstrate that such biologically guided surgical integration translates into meaningful differences in OS outcomes, underscoring the impact of risk-adapted surgical decision-making on long-term prognosis (49, 54, 66–68).

**Table 6:** Overall survival (OS) outcomes in pediatric adrenal neuroblastoma (NB).

Author (Year)	Study Design	Population	Main Findings in Overall Survival (OS)
Temple et al. (2021) (66)	Surgical retrospective cohort	76 NB/GNB	Infiltration of adjacent organs associated with worse OS (HR=8.90; p=0.007).
Gabra et al. (2022) (67)	Surgical multicenter (SIOPEN)	222 NB (54% adrenal); MIS cohort	10-year OS 91.8%. Extent of resection did not impact OS. MYCN amplification, age >18 months, and high-risk status determined OS.
Oesterreich et al. (2022) (54)	Surgical single center	17 laparoscopic adrenal NB	OS 86%; EFS 75%. No surgery-related mortality.
Bender et al. (2023) (68)	Cooperative study (COG)	NB <3 years (including adrenal)	5-year OS up to 94% after therapeutic de-escalation. Prognosis primarily driven by tumor biology.
He et al. (2024) (49)	Surgical single center	571 retroperitoneal NB	Perioperative mortality 0.53%. IDRFs associated with higher morbidity without demonstrated direct impact on OS.

GNB = Ganglioneuroblastoma; SIOPEN = International Society of Paediatric Oncology European Neuroblastoma Group; MIS = Minimally Invasive Surgery; EFS = Event-Free Survival; COG = Children's Oncology Group; IDRFs = Image-Defined Risk Factors; HR = Hazard Ratio; OS = Overall Survival.

This review presents limitations inherent to its narrative design. The methodological heterogeneity of included studies, predominantly retrospective and single center, limits direct comparability among series. Careful patient selection in minimally invasive surgery studies may overestimate perioperative benefits. Furthermore, the absence of randomized clinical trials comparing extent of resection and operative approach precludes definitive conclusions regarding oncologic superiority. Nevertheless, qualitative synthesis allows integration of contemporary evidence and delineation of consistent trends in the surgical management of pediatric adrenal NB.

## CONCLUSIONS

Surgical treatment of pediatric adrenal NB remains a strategic component of multimodal management, with its indication and extent modulated by a complex interaction between biological risk stratification, radiologic anatomical characteristics, and response to systemic therapy. It thus constitutes not merely a technical intervention, but a prognostic-modulating element within the multimodal therapeutic framework.

In low-risk tumors, complete resection represents a potentially curative strategy, whereas in intermediate- and high-risk groups, surgery assumes an integrative role in local disease control, prioritizing maximal macroscopic resection while preserving functional integrity and maintaining oncologic safety.

Careful evaluation of IDRFs guides technical planning and operative approach selection, maintaining open surgery as the standard for complex tumors and reserving minimally invasive approaches for carefully selected cases.

Surgical decision-making should therefore be individualized, biologically oriented, and grounded in principles that balance radicality and morbidity, with the aim of optimizing long-term oncologic outcomes.

.

## Data Availability

All data generated or analysed during this study are included in this published article

## ABBREVIATIONS

aOR: adjusted odds ratio
CCG: Children's Cancer Group
EFS: Event-Free Survival
IDRFs: Image-Defined Risk Factors
INRGSS: International Neuroblastoma Risk Group Staging System
INSS: International Neuroblastoma Staging System
INSRF: International Neuroblastoma Surgical Reporting Form
MIS: Minimally Invasive Surgery
NB: Neuroblastoma
OS: Overall Survival
PRA: Posterior Retroperitoneoscopic Adrenalectomy
PRISMA: Preferred Reporting Items for Systematic Reviews and Meta-Analyses
TLA: Transperitoneal Laparoscopic Adrenalectomy
